# A network analysis in adolescent anorexia nervosa exploring the connection between both patient and carer reactions and outcome

**DOI:** 10.1002/erv.2933

**Published:** 2022-06-19

**Authors:** Alessio Maria Monteleone, Giammarco Cascino, Laura Salerno, Ulrike Schmidt, Nadia Micali, Valentina Cardi, Janet Treasure

**Affiliations:** ^1^ Department of Psychiatry University of Campania “Luigi Vanvitelli” Naples Italy; ^2^ Department of Medicine Surgery and Dentistry ‘Scuola Medica Salernitana’ Section of Neurosciences University of Salerno Salerno Italy; ^3^ Department of Psychology, Educational Sciences and Human Movement University of Palermo Palermo Italy; ^4^ Department of Psychological Medicine Section of Eating Disorders Institute of Psychiatry, Psychology and Neuroscience King's College London London UK; ^5^ Department of Psychiatry Faculty of Medicine University of Geneva Geneva Switzerland; ^6^ UCL Great Ormond Street Institute of Child Health University College London London UK; ^7^ Department of General Psychology University of Padova Padova Italy

**Keywords:** anorexia nervosa, carers, depression, family intervention, network analysis

## Abstract

**Objective:**

This paper used network analysis to test the associations between eating disorder‐related psychopathology and carers’ responses to anorexia nervosa symptoms in adolescents. Additionally, the prognostic value of central and bridge network nodes was explored.

**Method:**

This is a secondary analysis of a three‐armed randomised‐controlled‐trial of adolescents with anorexia nervosa (*n* = 149) and their primary carer (*n* = 149) who were allocated to either treatment as usual (TAU), or one of two versions of a carer skills intervention (ECHO) added to TAU. A network analysis was run in the full sample. The prognostic role of central and bridge nodes was tested through multiple regression analyses.

**Results:**

Carers’ depression and emotional over‐involvement, as well as patients' depression showed the highest strength centrality. Patients' depression and carers’ accommodation exhibited the highest bridge expected influence. Across the full sample, and in the ECHO group, carers’ accommodation predicted patients' higher body mass index (BMI), while patients' depression predicted worse psychosocial functioning at 1‐year follow‐up. In the ECHO group, higher carers’ depression also predicted lower BMI.

**Conclusions:**

Carers’ accommodation and depression in both carers and patients were involved in the maintenance of psychopathology in adolescents with anorexia nervosa. Depression in both patients and carers is a potential treatment target for family interventions.

## Key points

- Accommodation is the carers’ variable most connected with adolescents' symptoms and is positively associated with adolescents' BMI at 1 year follow‐up.
- Depression is the adolescents' symptom most connected with carers’ functioning and predicts impaired psychosocial functioning at 1 year follow‐up.
- carers’ depression contributes to the maintenance of anorexia nervosa psychopathology and is negatively associated with adolescents' BMI at 1 year in ECHO treatment group.

**Abbreviations**
AESED: Accommodation and Enabling Scale for Eating Disorders
BEI: Bridge Expected Influence
BMI: Body Mass Index
CASK: Caregiver Skills Scale
CIA: Clinical Impairment Assessment
CS: Correlation Stability coefficient
DASS: Depression Anxiety and Stress Scales
ECHO: Experienced Carers Helping Others
EOI: Emotional Over Involvement
FBT: Family‐Based Treatyment
FQ: Family Questionnaire
MANTRA: Maudsley Model of Anorexia Nervosa Treatment for Adults
NICE: National Institute for Health and Care Excellence
OSSS: Oslo Social Support Scale
RCT: Randomised Controlled Trial
SEED: Short Evaluation of Eating Disorders
TAU: Treatment As Usual

## 1 INTRODUCTION

The inclusion of families in the treatment of adolescents with eating disorders is recommended by several clinical guidelines, including those formulated by the National Institute for Health and Care Excellence in the UK (NICE, 2004). Family‐based therapy in which parents implement refeeding has been the most widely disseminated treatment approach for adolescents with anorexia nervosa (Fisher et al., 2019). However, the remission rates at the end of treatment are unsatisfying (less than 50%) (Fisher et al., 2019).

Family‐based therapy originated as a behaviourally focussed adaptation of family therapy (Russell et al., 1987) and has been subsequently manualized and systematically studied in randomised control trials (Lock & Le Grange, 2019) which have shown its effectiveness in adolescents (NICE, 2017). However, not many predictors of clinical outcomes have been found, above and beyond early response to treatment (Lock & Le Grange, 2019). Lock et al. (2005, 2006) reported that features such as single parent families and obsessive‐compulsive and perseverative thinking styles in youths were associated with the need for longer treatment. Interestingly, a recent study used a network analysis and examined the contribution of parental self‐efficacy and eating disorder symptoms on clinical outcomes in adolescents with anorexia nervosa (Hagan et al., 2021). Parental beliefs in their responsibility to feed the child, and the child's level of dietary restraint and discomfort with social eating predicted weight restoration at the end of treatment (Hagan et al., 2021). Central symptoms (such as desiring weight loss, dietary restraint and feeling fat) failed to predict the response to treatment. This suggests that parental and social factors may need to be included in models of treatment.

The cognitive interpersonal model for anorexia nervosa (Schmidt & Treasure, 2006; Treasure et al., 2020; Treasure & Schmidt, 2013) includes aspects of both carers and child functioning in explaining illness maintenance. The main proposal is that the eating disorder elicits strong emotional reactions and extreme behaviours in others, such as anger, hostility, criticism, despair, avoidance, aggressiveness or hyper‐protection. These extreme reactions are not only associated with worse outcomes for patients', but also with increased carers’ distress and burden. Furthermore, carers’ burden is accentuated if their appraisals about the illness are negative (Matthews et al., 2018; Ohara et al., 2016), and if there is a personal family history of an eating disorder (Stefanini et al., 2019) or reduced social support (Treasure & Nazar, 2016), but not if carers are suffering from other psychiatric diagnoses (Monteleone et al., 2021). The cognitive interpersonal model has been used to inform treatments of eating disorders, such as the Maudsley Model of Anorexia Nervosa Treatment for Adults, that is, MANTRA (Schmidt et al., 2014), and the Experienced Carers Helping Others (ECHO) approach (Rhind et al., 2014). Both encourage a focus on carers’ behaviours as a target some of the illness maintaining factors. In a randomised clinical trial of the ECHO intervention (including self‐help materials to optimise caregiving skills), carers of adolescents with anorexia nervosa reported that caregiving was less burdensome at the end of the intervention. Also, the need for inpatient treatment was reduced (Hodsoll et al., 2017). However, the change in carers’ distress after treatment did not predict patients' BMI and a deeper investigation of the association between carers’ pre‐treatment distress and patients' clinical outcomes has been suggested (Salerno, Tchanturia et al., 2016).

Network theory (Fried & Cramer, 2017; McNally, 2016) posits that disorders do not emerge from a latent cause, but that the dynamic, causal interactions among symptoms themselves constitute the disorder. It also identifies an “external field” composed of those processes that are not part of the psychopathology but which can trigger symptoms (Borsboom, 2017). Thus, network analysis can simultaneously assess the interplay between carers’ distress and behavioural and emotional responses to the illness and patients' symptoms.

The aim of this paper was to use a network analysis including measures of both parental and adolescent functioning to predict clinical outcomes in anorexia nervosa. Our hypothesis was that parental high expressed emotion (criticism and over‐involvement), and accommodation would bridge carers’ distress (anxious and depressive symptoms) with adolescent eating disorder‐related symptoms and would lead to poorer outcomes (worse psychosocial functioning due to the illness and lower BMI) at 1 year follow‐up.

## 2 METHODS

### 2.1 Participants

This study was a secondary analysis of data from a large three‐arm multisite randomised controlled trial (RCT) (Rhind et al., 2014) evaluating the effect of ECHO, a carer‐skills training intervention for carers of adolescents with anorexia nervosa (Rhind et al., 2014). The original sample included 149 patients (aged between 12 and 21 years) and up to three of their carers (*n* = 226). For this analysis, 149 patients and their primary carer only (*n* = 149) were considered. Patients included adolescents with anorexia nervosa, or suffering from an eating disorder not otherwise specified of the anorexia nervosa subtype, according to the fourth edition of the Diagnostic and Statistical Manual of Mental Disorders criteria (APA, 1994). The primary carer was defined as the person with the highest number of contact hours with the patient.

### 2.2 Protocol

Patients and carers were recruited from consecutive referrals from 38 specialised eating disorders outpatient services across the UK. After obtaining informed consent, participants were randomly allocated to receive: (a) treatment as usual only (TAU, n = 50), (b) treatment as usual plus ECHO (ECHO as self‐help; n = 49), or (c) treatment as usual plus ECHOc (ECHO intervention supplemented by telephone guidance and therefore delivered as guided self‐help; n = 50). TAU was delivered by the participating centres and consisted of outpatient treatment as recommended by the National Institute for Health and Care Excellence's guidelines (NICE, 2004) for the treatment of anorexia nervosa in adolescents and young adults. The ECHO intervention is based on the cognitive interpersonal maintenance model of eating disorders (Goddard et al., 2011; Schmidt & Treasure, 2006). It combines psychoeducation with a skills training approach and consists of a book (Treasure et al., 2007) and theoretical and practical DVDs (Sepulveda et al., 2008). ECHOc is characterised by the additional use of telephone coaching sessions with individuals with lived or professional experience of eating disorders, who had been trained to use motivational interviewing. For this study, the ECHO and ECHOc groups were combined (*n* = 99) (Hodsoll et al., 2017). carers’ and patients' data were collected at baseline (referral to the outpatient service) and follow‐up (six and 12 months) (Rhind et al., 2014). The detailed protocol of the RCT is available elsewhere (Rhind et al., 2014).

### 2.3 Patients' measures

Single items from the Short Evaluation of Eating Disorders (SEED) (Bauer et al., 2005) were used at baseline to evaluate core eating disorder symptoms (i.e. drive to restriction, fear of weight gain, exercise and purging behaviours) over the previous week.

The Depression, Anxiety and Stress Scales (DASS‐21) (Lovibond & Lovibond, 1995) questionnaire was used at baseline to measure patients' psychological distress. The DASS‐21 is a 21‐item self‐report measure assessing depression, anxiety, and stress over the previous week using a 4‐point Likert scale (ranging from 0 = “Did not apply to me at all” to 3 = “Applied to me very much or most of the time”). The DASS‐21 Depression and Anxiety subscales were used in the present study and they show good to excellent internal consistency in the present study (Cronbach's α = 0.926 and 0.832 for depression and anxiety subscales, respectively).

The Clinical Impairment Assessment 3.0 (CIA) (Bohn et al., 2008) was used at baseline and at follow‐up to measure eating disorder‐related impairment of psychosocial functioning. The CIA is a 16‐item scale, and each item is rated on a four‐point Likert scale (ranging from 0 = “Not at all” to 3 = “A lot”). Higher scores indicate greater impairment. The CIA shows excellent internal consistency in the present study (Cronbach's α = 0.932 and 0.960 at baseline and at 1 year follow‐up, respectively).

Body Mass Index (BMI): BMI (weight/height^2^) was obtained from a medical assessment at 1 year follow‐up.

### 2.4 Carers’ measures

The Depression, Anxiety and Stress Scales (DASS‐21) (Lovibond & Lovibond, 1995) questionnaire was used at baseline to measure carers’ psychological distress. See earlier discussion for details about this instrument (i.e., Patients' measures subsection). The DASS‐21 depression and anxiety scores were used in the present study and they demonstrated good to excellent internal consistency (Cronbach's α = 0.908 and 0.827 for depression and anxiety subscale, respectively).

The Family Questionnaire (FQ) (Wiedemann et al., 2002) was used at baseline to measure carers’ expressed emotion, which measures carers’ attitudes and behaviours towards a relative with an illness. The FQ consists of 20 items and each item is rated on a four‐point Likert scale (ranging from 1 = “never/very rarely” to 4 = “very often”). The FQ has two subscales: critical comments, and emotional over involvement (EOI). Higher scores indicate greater levels of expressed emotion. Both subscales showed acceptable to good internal consistency in this study (Cronbach's α = 0.892 and 0.772 for criticism and EOI subscale, respectively).

The Accommodation and Enabling Scale for Eating Disorders (AESED) (Sepulveda et al., 2009) was used at baseline to measure carers’ accommodation to eating disorder symptoms (e.g., avoidance and modifying routines, providing reassurance, accepting rituals around meals, turning a blind eye to unwanted behaviours and allowing family functioning to be controlled). The AESED is a 33‐item scale rated on five‐point Likert scale (ranging from 0 = “never” to 4 = “every day”). A high AESED score is associated with high family accommodation to eating disorder symptoms. The AESED total score was used in the present study, and showed excellent internal consistency (Cronbach's α = 0.926).

The Caregiver Skills Scale (CASK) (Hibbs et al., 2015) was used at baseline to measure carers’ skills (Goddard et al., 2011; Schmidt & Treasure, 2006). The CASK has 27 items, rated on a visual analogue scale with anchors at 0 and 100, with higher values representing higher self‐assessed skill‐levels. The CASK total score used in the present study had excellent internal consistency (Cronbach's α = 0.908).

The Oslo Social Support Scale (OSSS‐3) (Kocalevent et al., 2018) was used at baseline to measure perceived social support (covering support from family, friends and neighbourhood). The OSSS‐3 consists of three items; the first item is rated on a four‐point Likert scale from one to four and the other two items were rated on a five‐point Likert scale from one to five, with item‐specific response options. High values represent strong levels of social support. The OSSS‐3 shows acceptable internal consistency in the present study (Cronbach's α = 0.752).

### 2.5 Analyses

Analyses of internal consistency and descriptive statistics were conducted in SPSS v. 22. Network analysis was conducted using R (R Core Team, 2016), version 3.4.4, using the *qgraph* package. The network included the overall patient population (*n* = 149) at baseline, before receiving the intervention. Patients' nodes were the core eating disorder symptoms (drive to restriction, fear of weight gain, exercise and purging behaviours from the SEED), eating disorder‐related impairment on psychosocial functioning (CIA), and depressive and anxiety symptoms (DASS‐21). carers’ nodes included carers’ responses to the illness, that is, caregiver skills (CASK), emotional over‐involvement and criticism (FQ), accommodation (AESED), social support (OSLO) and perceived distress (anxiety and depression from the DASS‐21).

According to the methodology described by Epskamp et al. (2018), a partial correlational network analysis was performed. The strength centrality index was measured and plotted. Following the recommendations by Epskamp et al. (2018), the stability of the network was estimated using the bootnet package (Epskamp et al., 2016). First, the Correlation Stability (CS) coefficient was estimated. This corresponds to the maximum proportion of the population that could be dropped so that the correlation between the re‐calculated indices of the obtained networks and those of the original network would equal or be higher than 0.7. Epskamp and Fried (2018) suggested that 0.25 is the minimum cut‐off to consider the network reliable. The accuracy of edge‐weights was calculated by drawing bootstrapped confidence intervals using nonparametric bootstrapping (nboots = 2500). The bridge symptoms (i.e., those playing a primary role in connecting two or more symptom communities (Jones et al., 2021)) were selected. The bridge function from the *networktools* package (Jones, 2017) was employed to estimate bridge centrality. The bridge expected influence, which is the raw sum of the values (+ or −) of all edges that connect a node to all nodes that are not part of the same community (Levinson et al., 2018), was calculated. A high bridge expected value means that the node is strongly and positively connected to other nodes included in other communities. An a priori definition of communities was applied, and two communities were identified: one representing patients' symptoms (eating disorder‐related psychopathology, depressive and anxiety symptoms, and clinical impairment due to the illness), and the other including carers’ reactions (caregiving skills, emotional over‐involvement, criticism, accommodation, perceived social support, and depression and anxiety).

### 2.6 Regression analyses

Multiple regression analyses were carried out to test the prognostic utility of central and bridge nodes in the pre‐treatment network. These nodes were included as predictors. Adolescents' BMI and eating disorder‐related impairment of psychosocial functioning at 1 year follow‐up were entered as outcomes in these models. Regression analyses were calculated across the full sample (*n* = 121), and then separately for TAU (TAU *n* = 37) and TAU plus ECHO (*n* = 84) groups. Neither patients' BMI, nor age, or duration of illness were entered as covariates in the models because the inclusion criteria limited the variation in age, illness duration and BMI. A *t*‐test for independent samples was used to compare clinical characteristics between patients included in the follow‐up and those who discontinued.

## 3 RESULTS

Demographic and clinical characteristics of the sample are reported in Table 1.

**TABLE 1:** Demographic and clinical characteristics of the sample

	Patients (*n* = 149)	Primary Carer (*n* = 149)
Age, *M* (SD)	16.89 (2.13)	48.09 (5.99)
Gender, *n* (%) females	137 (91.9)	142 (95.3)
Years in education, *M* (SD)	11.76 (1.99)	15.45 (3.67)
BMI on admission (kg/m^2^), *M* (SD)	16.85 (2.23)	‐
Primary diagnosis, *n* (%)		
Anorexia nervosa	112 (75.2)	‐
Eating disorders not otherwise specified with restricting features	37 (24.8)	‐
Illness duration (months), *M* (SD)	22.35 (22.37)	‐
Carer type, *n* (%)		
Mother	‐	139 (93.3)
Father	‐	7 (4.7)
Grandmother	‐	1 (0.7)
Sibling	‐	2 (1.3)
Employment status, *n* (%)		
Employed (full time/part time)	12 (8.0)	103 (69.1)
Unemployed/retired/sick leave/student	136 (91.3)	32 (21.5)
Other/missing	1 (0.7)	14 (9.4)
Marital status, *n* (%)		
Married/living together/in a relationship	22 (14.7)	112 (75.2)
Single/divorced/separated/widowed	119 (79.9)	35 (23.5)
Missing	8 (5.4)	2 (1.3)

At the time of admission to the study the adolescents with anorexia nervosa (female (91.9%) had a BMI of 16.8 (SD = 2.2) and were aged 16.9 (SD = 2.1). Mothers (married or living with a partner (75.2%) were most often (93.3%) the primary carer and (69.1%) were also employed.

### 3.1 Network analysis

**FIGURE 1:**
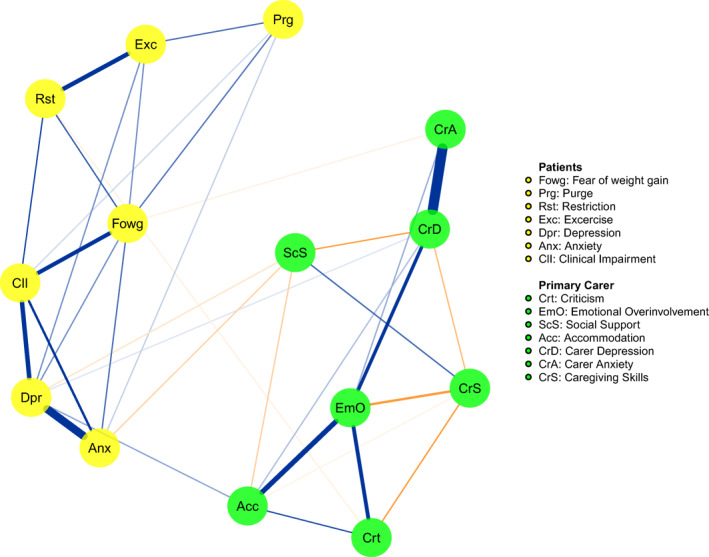
Estimated network of overall patient population (*n* = 149) at baseline

**FIGURE 2:**
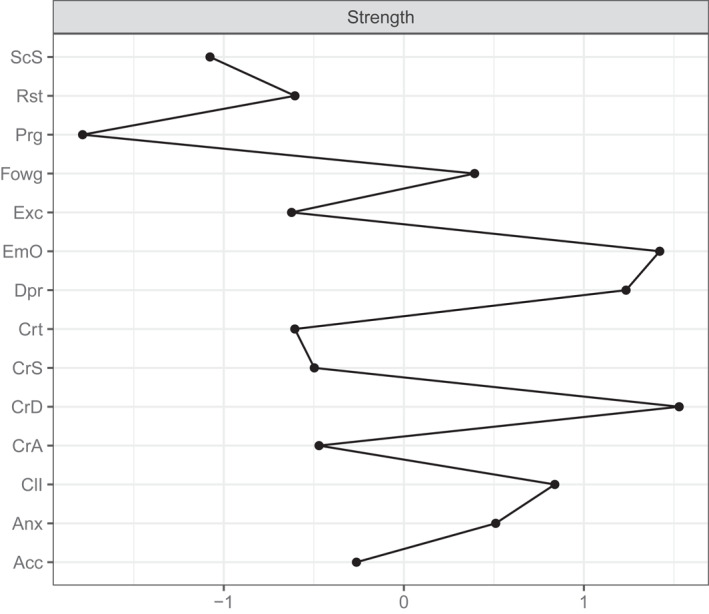
Plot of the strength centrality indices of each network node. Acc, carer's accommodation; Anx, patient's anxiety; CII, patient's clinical impairment; CrA, carer's anxiety; CrD, carer's depression; CrS, caregiving skills; Crt, carer's criticism; Dpr, patient's depression; EmO, carer's emotional over‐involvement; Exc, patient's exercise; Fowg, patient's fear of weight gain; Prg, patient's purging; Rst, patient's restriction; Scs, carer's social support

**FIGURE 3:**
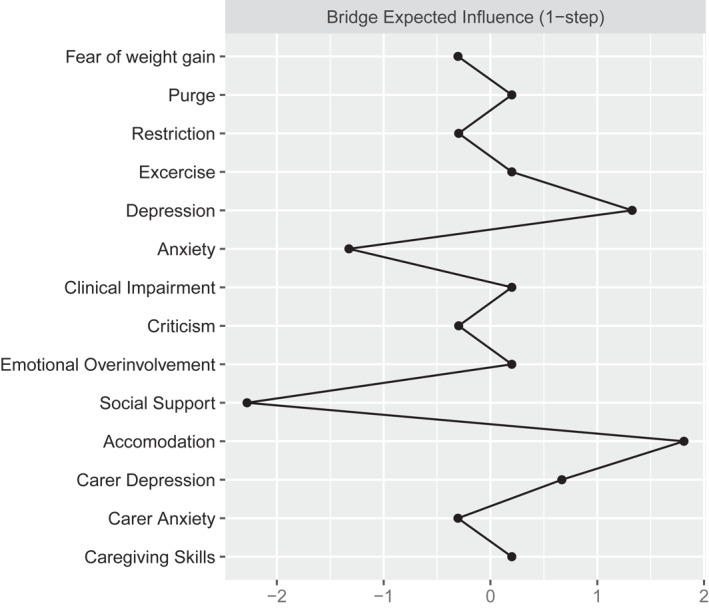
Plot of the bridge expected influence of each network node

The pre‐treatment network is reported in Figure 1; strength centrality indices are plotted in Figure 2. The network was stable (the CS of the network was 0.36 for strength). Carers’ depression (*M* = 1.05) and emotional over‐involvement (*M* = 1.02), and patients' depression (*M* = 0.98) emerged as the most central nodes in the network. According to the strength centrality difference test, the strength centrality index of these nodes was significantly higher than 72% of all other nodes estimates. Bridge expected influence of network nodes are plotted in Figure 3. Patients' depression (BEI = 0.07) and carers’ accommodation towards the illness (BEI = 0.05) were the nodes with the highest bridge expected influence.

### 3.2 Regression analyses

Multiple regression analyses were run first across the full sample (*n* = 121), then in each treatment group separately. Patients' depression, and carers’ accommodation towards the illness, depression and emotional over‐involvement were included as predictors, while BMI and clinical impairment at 1 year were included as outcomes in different models. Across the full sample, carers’ accommodation towards the illness positively predicted patients BMI at 1 year (*ß* = 0.20; *p* = 0.04). Patients' depression predicted eating disorder‐related impairment of psychosocial functioning at 1 year (*ß* = 0.27; *p* = 0.008). No significant predictors were identified in the TAU group. In the ECHO group, carers’ accommodation towards the illness was positively associated with patients' BMI at 1 year (*ß* = 0.23; *p* = 0.02), while carers’ depression was negatively associated with patients' BMI at 1 year (*ß* = −0.22; *p* = 0.04). In the ECHO group, patients' depression was positively associated with eating disorder‐related impairment of psychosocial functioning at 1 year follow‐up (*ß* = 0.31; *p* = 0.009).

Comparison of patients who underwent the follow‐up assessment (*n* = 121) with those who did not (*n* = 28) revealed no significant differences in BMI, illness duration, age, patients' variables (eating disorder symptoms, eating disorder‐related impairment on psychosocial functioning, depressive and anxiety symptoms) and carer's variables (caregiver skills, emotional over‐involvement and criticism, accommodation, social support, and perceived distress). Thus, the subgroup of follow‐up patients included in the regression analysis was representative of the all sample.

## 4 DISCUSSION

We used a network analysis to test the associations between carers’ emotional and behavioural reactions to the eating disorder and patients' clinical outcomes. Patients' depression, and carers’ depression and emotional over‐involvement were the most central nodes of the network; patients' depression and carers’ accommodation were the nodes with the highest bridge function and predicted treatment outcomes across the full sample. The prognostic role of these variables was confirmed in the ECHO group alone, in which carers’ depression predicted treatment outcomes.

Following recent recommendations for conducting network analysis in studies of eating disorders (Monteleone & Cascino, 2021), a highly inclusive network was calculated including patients' eating and affective symptoms, and carers’ emotional and behavioural reactions to the illness (Treasure & Nazar, 2016). The resulting network was stable and reproducible according to the criteria proposed by Epskamp and Fried (2018). In line with the cognitive interpersonal model of anorexia nervosa (Treasure & Schmidt, 2013), findings indicated that carers’ distress (depressive and anxious symptoms) and reactions to the illness were associated with the illness psychopathology, and likely contribute to its maintenance. Only one previous study (Hagan et al., 2021) has included carers and patients' variables in a network of adolescents with anorexia nervosa. However, that study only considered one caregiving variable, that is, parental self‐efficacy, and no measures of affective symptoms or other caregiving behaviours.

Our study hypothesis was partially confirmed. Carers’ accommodation was the carers’ variable most connected with patients' symptoms, thus bridging carers’ distress (anxious and depressive symptoms) with the adolescent symptoms. These results and the centrality of carers’ depression align with findings in the literature noting the association between carers’ depression, accommodation and eating disorder symptoms.

Negative emotions, such as guilt and anxiety are prevalent among carers experiencing suboptimal outcomes from family‐based therapy for eating disorders (Wufong et al., 2019). Carers recognised that accommodating behaviours reduced their self‐efficacy and interfered with the goal of recovery (Fox & Whittlesea, 2017). Another study found that carers of more severely ill adolescents with anorexia nervosa reported significantly greater levels of depression and expressed emotions and these variables were interrelated (Schwarte et al., 2017).

The association between carers’ depression and patients' eating disorder‐related symptoms were confirmed in the ECHO group, where carers’ depression at baseline was associated with lower patients' BMI at 1‐year follow‐up. This is consistent with previous findings displaying that maternal guilt at baseline was associated with eating disorder‐related symptoms at the end of treatment in adolescents with anorexia nervosa (Dennhag et al., 2019). In a previous analysis of these data, it was found that carers receiving the ECHO skill‐based training intervention reported less distress (small effects) at follow‐up compared to the control group (Hodsoll et al., 2017). Similarly, patients in the ECHO group reported lower distress (small effects), fewer hospital admissions and a trend for higher BMI at follow‐up (Hodsoll et al., 2017). Altogether, these findings generate interesting hypotheses, such as that the effectiveness of the ECHO treatment might be due to its effects on carers’ depression or that carers with high levels of depression might benefit more from the intervention. These suggestions may be further reinforced by the evidence that no significant associations were seen in the TAU group, although the low size of this sample may have affected this result.

The network analysis in this study not only demonstrated the importance of carers’ depression, but also the centrality of patients' depression in the network. This mirrors findings from a network analysis conducted in a large sample of adolescents with anorexia nervosa which found that depression featured strongly in the central network node (Monteleone et al., 2019). Studies involving adults with anorexia nervosa have also found that comorbidity with depression has an adverse effect on clinical outcomes (Ambwani et al., 2020; Keshishian et al., 2021). Interestingly, patients' depression rather than eating disorder symptoms connected with carers’ functioning. This is an interesting, yet an under‐investigated link which might shed further light on the maintenance of eating disorder symptoms through patient‐ and carer‐related variables.

Across the full sample patients' depression predicted impaired psychosocial functioning at 1 year follow‐up, corroborating similar findings from the same database (Salerno, Tchanturia et al., 2016).

A somewhat puzzling finding was the positive association between carers’ accommodating behaviours and patients' BMI at follow‐up. This is not consistent with the predictions from the cognitive interpersonal model of anorexia nervosa (Treasure & Schmidt, 2013) although accommodating behaviours are an understandable reaction to the distress of others. A recent review suggested that emotional over‐involvement, a construct including accommodating behaviours (Wamboldt et al., 2000), might have some benefits across a wide range of psychiatric and medical disorders (Rienecke, 2020). This may occur because carers who are accommodating may validate their child's distress, and the children can perceive the carer as helping them and being on their side. It may be that only if accommodating behaviours are prolonged or excessive they may serve to embed unhelpful habits (Schmidt & Treasure, 2006). Also, there may be an interaction between parents. For example, a previous study found that patient's distress was worse when both mother and father were high in accommodation (Salerno, Lo Coco et al., 2016). It is possible to hypothesise that a “good enough dose” of emotional over‐involvement, including accommodating behaviours, might favour positive clinical change. Overall, future studies are needed to explore whether the effectiveness of family interventions occurs by changing accommodating processes or only in the presence of these behaviours that may be associated with a heightened family involvement towards the treatment.

### 4.1 Strengths and limitations

The main limitations of this study are that only data from one carer were analysed, and that the clinical sample included individuals with short duration of illness and moderate illness severity, which limits the generalisability of the findings. A further limitation is the lack of inclusion in the network of factors that had negatively predicted outcome from FBT (Lock et al., 2005) such as obsessive‐compulsive symptoms and marital status of caregivers. Despite these limitations, the study included a wide variety of patient and carer variables in the network analysis, and it assessed the prognostic role of central and bridge nodes. Interestingly, the nodes with the highest centrality and bridge function predicted treatment outcome in the overall group of patients. This confirms that network analysis might have prognostic utility (Fried & Cramer, 2017), also in the field of eating disorders (for a review see Monteleone & Cascino, 2021).

### 4.2 Clinical implications

These findings highlight the importance of monitoring and addressing depressive symptoms in both patients with anorexia nervosa and their carers. Encouraging evidence from eating disorder family therapies demonstrates that these therapies are effective in reducing depression and negative aspects of caregiving in family members (Baudinet et al., 2021). Furthermore, the current data generate the hypothesis that carers’ accommodating behaviours might be helpful to some extent in the treatment of adolescents with anorexia nervosa.

## CONFLICTS OF INTEREST

Janet Treasure received royalties for books on skills based caring from Routledge. Other authors have nothing to declare.

## CLINICAL TRIAL REGISTRATION NUMBER

ISRCTN83003225.

## Data Availability

The data that support the findings of this study are available from the corresponding author upon reasonable request.

## References

[erv2933-bib-0001] Ambwani, S. , Cardi, V. , Albano, G. , Cao, L. , Crosby, R. D. , Macdonald, P. , Schmidt, U. , & Treasure, J. (2020). A multicenter audit of outpatient care for adult anorexia nervosa: Symptom trajectory, service use, and evidence in support of “early stage” versus “severe and enduring” classification. International Journal of Eating Disorders, 53, 1337–1348. 10.1002/eat.23246 32064663

[erv2933-bib-0002] APA . (1994). American Psychiatric Association. Diagnostic and statistical manual of mental disorders (4th ed.). American Psychiatric Association.

[erv2933-bib-0003] Baudinet, J. , Eisler, I. , Dawson, L. , Simic, M. , & Schmidt, U. (2021). Multi‐family therapy for eating disorders: A systematic scoping review of the quantitative and qualitative findings. International Journal of Eating Disorders, 54(12), 2095–2120. 10.1002/EAT.23616 34672007 PMC9298280

[erv2933-bib-0004] Bauer, S. , Winn, S. , Schmidt, U. , & Kordy, H. (2005). Construction, scoring and validation of the short evaluation of eating disorders (SEED). European Eating Disorders Review, 13(3), 191–200. 10.1002/ERV.637

[erv2933-bib-0005] Bohn, K. , Doll, H. A. , Cooper, Z. , O'Connor, M. , Palmer, R. L. , & Fairburn, C. G. (2008). The measurement of impairment due to eating disorder psychopathology. Behaviour Research and Therapy, 46(10), 1105–1110. 10.1016/J.BRAT.2008.06.012 18710699 PMC2764385

[erv2933-bib-0006] Borsboom, D. (2017). A network theory of mental disorders. World Psychiatry, 16(1), 5–13. 10.1002/wps.20375 28127906 PMC5269502

[erv2933-bib-0007] Dennhag, I. , Henje, E. , & Nilsson, K. (2019). Parental caregiver burden and recovery of adolescent anorexia nervosa after multi‐family therapy. Eating Disorders, 29(5), 463–479. 10.1080/10640266.2019.1678980 31617832

[erv2933-bib-0008] Epskamp, S. , Borsboom, D. , & Fried, E. I. (2018). Estimating psychological networks and their accuracy: A tutorial paper. Behavior Research Methods, 50(1), 195–212. 10.3758/s13428-017-0862-1 28342071 PMC5809547

[erv2933-bib-0009] Epskamp, S. , & Fried, E. I. (2018). A tutorial on regularized partial correlation networks. Psychological Methods, 23(4), 617–634. 10.1037/met0000167 29595293

[erv2933-bib-0010] Epskamp, S. , Maris, G. K. J. , Waldorp, L. J. , & Borsboom, D. (2016). Network psychometrics. In The Wiley Handbook of psychometric testing: A multidisciplinary reference on survey, scale and test development (Vol. 2, pp. 953–986). https://arxiv.org/abs/1609.02818v2

[erv2933-bib-0011] Fisher, C. A. , Skocic, S. , Rutherford, K. A. , Hetrick, S. E. , & Group, C. C. M. D. (2019). Family therapy approaches for anorexia nervosa. Cochrane Database of Systematic Reviews, 5. 10.1002/14651858.CD004780.PUB4 PMC6497182 31041816

[erv2933-bib-0012] National Guideline Alliance, U . (2017). Eating disorders: Recognition and treatment. National Institute for Health and Care Excellence (NICE). http://www.nice.org.uk/guidance/ng69 28654225

[erv2933-bib-0013] Fox, J. R. E. , & Whittlesea, A. (2017). Accommodation of symptoms in anorexia nervosa: A qualitative study. Clinical Psychology & Psychotherapy, 24(2), 488–500. 10.1002/CPP.2020 27312350

[erv2933-bib-0014] Fried, E. I. , & Cramer, A. O. J. (2017). Moving forward: Challenges and directions for psychopathological network theory and methodology. Perspectives on Psychological Science, 12(6), 999–1020. 10.1177/1745691617705892 28873325

[erv2933-bib-0015] Goddard, E. , Macdonald, P. , Sepulveda, A. R. , Naumann, U. , Landau, S. , Schmidt, U. , & Treasure, J. (2011). Cognitive interpersonal maintenance model of eating disorders: Intervention for carers. The British Journal of Psychiatry: Journal of Mental Science, 199(3), 225–231. 10.1192/BJP.BP.110.088401 21727233

[erv2933-bib-0016] Hagan, K. E. , Matheson, B. E. , Datta, N. , L’Insalata, A. M. , Onipede, Z. A. , Gorrell, S. , Mondal, S. , Bohon, C. M. , Grange, D. Le , & Lock, J. D. (2021). Understanding outcomes in family‐based treatment for adolescent anorexia nervosa: A network approach. Psychological Medicine, 1–12. 10.1017/S0033291721001604 PMC8820974 33952357

[erv2933-bib-0017] Hibbs, R. , Rhind, C. , Salerno, L. , Lo Coco, G. , Goddard, E. , Schmidt, U. , Micali, N. , Gowers, S. , Beecham, J. , MacDonald, P. , Todd, G. , Campbell, I. , & Treasure, J. (2015). Development and validation of a scale to measure caregiver skills in eating disorders. International Journal of Eating Disorders, 48(3), 290–297. 10.1002/EAT.22362 25351932

[erv2933-bib-0018] Hodsoll, J. , Rhind, C. , Micali, N. , Hibbs, R. , Goddard, E. , Nazar, B. P. , Schmidt, U. , Gowers, S. , Macdonald, P. , Todd, G. , Landau, S. , & Treasure, J. (2017). A pilot, multicentre pragmatic randomised trial to explore the impact of carer skills training on carer and patient behaviours: Testing the cognitive interpersonal model in adolescent anorexia nervosa. European Eating Disorders Review: The Journal of the Eating Disorders Association, 25(6), 551–561. 10.1002/ERV.2540 28948663

[erv2933-bib-0019] Jones, P. J. (2017). networktools: Assorted tools for identifying important nodes in networks. R package version 1.0.0

[erv2933-bib-0020] Jones, P. J. , Ma, R. , & McNally, R. J. (2021). Bridge centrality: A network approach to understanding comorbidity. Multivariate Behavioral Research, 56(2), 353–367. 10.1080/00273171.2019.1614898 31179765

[erv2933-bib-0021] Keshishian, A. C. , Tabri, N. , Becker, K. R. , Franko, D. L. , Herzog, D. B. , Thomas, J. J. , & Eddy, K. T. (2021). Comorbid depression and substance use prospectively predict eating disorder persistence among women with anorexia nervosa and bulimia nervosa. Journal of Behavioral and Cognitive Therapy, 31(4), 309–315. 10.1016/J.JBCT.2021.09.003

[erv2933-bib-0022] Kocalevent, R. D. , Berg, L. , Beutel, M. E. , Hinz, A. , Zenger, M. , Härter, M. , Nater, U. , & Brähler, E. (2018). Social support in the general population: Standardization of the Oslo social support scale (OSSS‐3). BMC Psychology, 6(1), 1–8. 10.1186/S40359-018-0249-9/TABLES/7 30016997 PMC6050647

[erv2933-bib-0023] Levinson, C. A. , Brosof, L. C. , Vanzhula, I. , Christian, C. , Jones, P. , Rodebaugh, T. L. , Langer, J. K. , White, E. K. , Warren, C. , Weeks, J. W. , Menatti, A. , Lim, M. H. , & Fernandez, K. C. (2018). Social anxiety and eating disorder comorbidity and underlying vulnerabilities: Using network analysis to conceptualize comorbidity. International Journal of Eating Disorders, 51(7), 693–709. 10.1002/eat.22890 30102777

[erv2933-bib-0024] Lock, J. , Agras, W. S. , Bryson, S. , & Kraemer, H. C. (2005). A comparison of short‐ and long‐term family therapy for adolescent anorexia nervosa. Journal of the American Academy of Child & Adolescent Psychiatry, 44(7), 632–639. 10.1097/01.CHI.0000161647.82775.0A 15968231

[erv2933-bib-0025] Lock, J. , Couturier, J. , Bryson, S. , & Agras, S. (2006). Predictors of dropout and remission in family therapy for adolescent anorexia nervosa in a randomized clinical trial. International Journal of Eating Disorders, 39(8), 639–647. 10.1002/EAT.20328 16927385

[erv2933-bib-0026] Lock, J. , & Le Grange, D. (2019). Family‐based treatment: Where are we and where should we be going to improve recovery in child and adolescent eating disorders. International Journal of Eating Disorders, 52(4), 481–487. 10.1002/EAT.22980 30520532

[erv2933-bib-0027] Lovibond, P. F. , & Lovibond, S. H. (1995). The structure of negative emotional states: Comparison of the depression anxiety stress scales (DASS) with the beck depression and anxiety inventories. Behaviour Research and Therapy, 33(3), 335–343. 10.1016/0005-7967(94)00075-U 7726811

[erv2933-bib-0028] Matthews, A. , Lenz, K. R. , Peugh, J. , Copps, E. C. , & Peterson, C. M. (2018). Caregiver burden and illness perceptions in caregivers of medically hospitalized youth with anorexia nervosa. Eating Behaviors, 29, 14–18. 10.1016/J.EATBEH.2018.01.003 29413819

[erv2933-bib-0029] McNally, R. J. (2016). Can network analysis transform psychopathology? Behaviour Research and Therapy, 86, 95–104. 10.1016/j.brat.2016.06.006 27424882

[erv2933-bib-0030] Monteleone, A. M. , & Cascino, G. (2021). A systematic review of network analysis studies in eating disorders: Is time to broaden the core psychopathology to non specific symptoms. European Eating Disorders Review, 29(4), 531–547. 10.1002/ERV.2834 33942439 PMC8251923

[erv2933-bib-0031] Monteleone, A. M. , Mereu, A. , Cascino, G. , Castiglioni, M. C. , Marchetto, C. , Grasso, M. , Pontillo, M. , Pisano, T. , Vicari, S. , & Zanna, V. (2021). Coping with adolescents affected by anorexia nervosa: The role of parental personality traits. Frontiers in Psychology, 12, 2586. 10.3389/FPSYG.2021.678745/BIBTEX PMC8292794 34305733

[erv2933-bib-0032] Monteleone, A. M. , Mereu, A. , Cascino, G. , Criscuolo, M. , Castiglioni, M. C. , Pellegrino, F. , Patriciello, G. , Ruzzi, V. , Monteleone, P. , Vicari, S. , & Zanna, V. (2019). Re‐conceptualization of anorexia nervosa psychopathology: A network analysis study in adolescents with short duration of the illness. International Journal of Eating Disorders, 52(11), 1263–1273. 10.1002/eat.23137 31313374

[erv2933-bib-0033] NICE . (2004). National Collaborating Centre for Mental Health. In Eating Disorders: Core Interventions in the treatment and management of anorexia nervosa, bulimia nervosa and related eating disorders. https://www.nice.org.uk/guidance/ng69 23346610

[erv2933-bib-0034] Ohara, C. , Komaki, G. , Yamagata, Z. , Hotta, M. , Kamo, T. , & Ando, T. (2016). Factors associated with caregiving burden and mental health conditions in caregivers of patients with anorexia nervosa in Japan. BioPsychoSocial Medicine, 10(1), 1–9. 10.1186/S13030-016-0073-5/TABLES/6 PMC4918134 27340430

[erv2933-bib-0035] R Core Team, R. (2016). A language and environment for statistical computing. R Foundation for Statistical Computing.

[erv2933-bib-0036] Rhind, C. , Hibbs, R. , Goddard, E. , Schmidt, U. , Micali, N. , Gowers, S. , Beecham, J. , Macdonald, P. , Todd, G. , Tchanturia, K. , & Treasure, J. (2014). Experienced Carers Helping Others (ECHO): Protocol for a pilot randomised controlled trial to examine a psycho‐educational intervention for adolescents with anorexia nervosa and their carers. European Eating Disorders Review: The Journal of the Eating Disorders Association, 22(4), 267–277. 10.1002/ERV.2298 24888426

[erv2933-bib-0037] Rienecke, R. D. (2020). Emotional overinvolvement with adolescents: A problematic construct? Current Treatment Options in Psychiatry, 7(2), 162–185. 10.1007/S40501-020-00205-Z/TABLES/1

[erv2933-bib-0038] Russell, G. F. M. , Szmukler, G. I. , Dare, C. , & Eisler, I. (1987). An evaluation of family therapy in anorexia nervosa and bulimia nervosa. Archives of General Psychiatry, 44(12), 1047–1056. 10.1001/ARCHPSYC.1987.01800240021004 3318754

[erv2933-bib-0039] Salerno, L. , Rhind, C. , Hibbs, R. , Micali, N. , Schmidt, U. , Gowers, S. , Macdonald, P. , Goddard, E. , Todd, G. , Lo Coco, G. , & Treasure, J. (2016). An examination of the impact of care giving styles (accommodation and skilful communication and support) on the one year outcome of adolescent anorexia nervosa: Testing the assumptions of the cognitive interpersonal model in anorexia nervosa. Journal of Affective Disorders, 191, 230–236. 10.1016/J.JAD.2015.11.016 26682492

[erv2933-bib-0040] Salerno, L. , Rhind, C. , Hibbs, R. , Micali, N. , Schmidt, U. , Gowers, S. , Macdonald, P. , Goddard, E. , Todd, G. , Tchanturia, K. , Lo Coco, G. , & Treasure, J. (2016). A longitudinal examination of dyadic distress patterns following a skills intervention for carers of adolescents with anorexia nervosa. European Child & Adolescent Psychiatry, 25(12), 1337–1347. 10.1007/S00787-016-0859-9 27161339

[erv2933-bib-0041] Schmidt, U. , & Treasure, J. (2006). Anorexia nervosa: Valued and visible. A cognitive‐interpersonal maintenance model and its implications for research and practice. British Journal of Clinical Psychology, 45(Pt 3), 343–366. http://www.ncbi.nlm.nih.gov/pubmed/17147101 17147101 10.1348/014466505x53902

[erv2933-bib-0042] Schmidt, U. , Wade, T. D. , & Treasure, J. (2014). The maudsley model of anorexia nervosa treatment for adults (MANTRA): Development, key features, and preliminary evidence. Journal of Cognitive Psychotherapy: International Quarterly, 28(1), 48–71, 10.1891/0889-8391.28.1.48 32759130

[erv2933-bib-0043] Schwarte, R. , Timmesfeld, N. , Dempfle, A. , Krei, M. , Egberts, K. , Jaite, C. , Fleischhaker, C. , Wewetzer, C. , Herpertz‐Dahlmann, B. , Seitz, J. , & Bühren, K. (2017). Expressed emotions and depressive symptoms in caregivers of adolescents with first‐onset anorexia nervosa‐A long‐term investigation over 2.5 years. European Eating Disorders Review: The Journal of the Eating Disorders Association, 25(1), 44–51. 10.1002/ERV.2490 27943533

[erv2933-bib-0044] Sepulveda, A. R. , Kyriacou, O. , & Treasure, J. (2009). Development and validation of the accommodation and enabling scale for eating disorders (AESED) for caregivers in eating disorders. BMC Health Services Research, 9(1), 1–13. 10.1186/1472-6963-9-171/TABLES/5 19775448 PMC2759929

[erv2933-bib-0045] Sepulveda, A. R. , Lopez, C. , Macdonald, P. , & Treasure, J. (2008). Feasibility and acceptability of DVD and telephone coaching‐based skills training for carers of people with an eating disorder. International Journal of Eating Disorders, 41(4), 318–325. 10.1002/EAT.20502 18176950

[erv2933-bib-0046] Stefanini, M. C. , Troiani, M. R. , Caselli, M. , Dirindelli, P. , Lucarelli, S. , Caini, S. , & Martinetti, M. G. (2019). Living with someone with an eating disorder: Factors affecting the caregivers' burden. Eating and Weight Disorders: EWD, 24(6), 1209–1214. 10.1007/S40519-018-0480-7 29368292

[erv2933-bib-0047] Treasure, J. , & Nazar, B. P. (2016). Interventions for the carers of patients with eating disorders. Current Psychiatry Reports, 18(2), 16. 10.1007/s11920-015-0652-3 26781554 PMC4718944

[erv2933-bib-0048] Treasure, J. , & Schmidt, U. (2013). The cognitive‐interpersonal maintenance model of anorexia nervosa revisited: A summary of the evidence for cognitive, socio‐emotional and interpersonal predisposing and perpetuating factors. Journal of Eating Disorders, 1(1), 13. 10.1186/2050-2974-1-13 24999394 PMC4081714

[erv2933-bib-0049] Treasure, J. , Smith, G. , & Crane, A. (2007). Skills‐based learning for caring for a loved one with an eating disorder: The new Maudsley method. Routledge Taylor & Francis Group. 10.4324/9780203945896

[erv2933-bib-0050] Treasure, J. , Willmott, D. , Ambwani, S. , Cardi, V. , Clark Bryan, D. , Rowlands, K. , & Schmidt, U. (2020). Cognitive interpersonal model for anorexia nervosa revisited: The perpetuating factors that contribute to the development of the severe and enduring illness. Journal of Clinical Medicine, 9(3), 630. 10.3390/jcm9030630 32120847 PMC7141127

[erv2933-bib-0051] Wamboldt, F. S. , Connor, W. O. , Wamboldt, M. Z. , Gavin, L. A. , & Klinnert, M. D. (2000). The five minute speech sample in children with asthma: Deconstructing the construct of expressed emotion. Journal of Child Psychology and Psychiatry, 41(7), 887–898. 10.1111/1469-7610.00676 11079431

[erv2933-bib-0052] Wiedemann, G. , Rayki, O. , Feinstein, E. , & Hahlweg, K. (2002). The family questionnaire: Development and validation of a new self‐report scale for assessing expressed emotion. Psychiatry Research, 109(3), 265–279. 10.1016/S0165-1781(02)00023-9 11959363

[erv2933-bib-0053] Wufong, E. , Rhodes, P. , & Conti, J. (2019). We don’t really know what else we can do: Parent experiences when adolescent distress persists after the Maudsley and family‐based therapies for anorexia nervosa. Journal of Eating Disorders, 7(1), 1–18. 10.1186/S40337-019-0235-5/FIGURES/1 30805186 PMC6373134

